# Temperature Effects on *Agrobacterium* Phytochrome Agp1

**DOI:** 10.1371/journal.pone.0025977

**Published:** 2011-10-17

**Authors:** Ibrahim Njimona, Tilman Lamparter

**Affiliations:** Botanical Institute, Karlsruhe Institute of Technology (KIT) Campus South, Karlsruhe, Germany; Research Institute for Children and the Louisiana State University Health Sciences Center, United States of America

## Abstract

Phytochromes are widely distributed biliprotein photoreceptors with a conserved N-terminal chromophore-binding domain. Most phytochromes bear a light-regulated C-terminal His kinase or His kinase-like region. We investigated the effects of light and temperature on the His kinase activity of the phytochrome Agp1 from *Agrobacterium tumefaciens*. As in earlier studies, the phosphorylation activity of the holoprotein after far-red irradiation (where the red-light absorbing Pr form dominates) was stronger than that of the holoprotein after red irradiation (where the far red-absorbing Pfr form dominates). Phosphorylation activities of the apoprotein, far red-irradiated holoprotein, and red-irradiated holoprotein decreased when the temperature increased from 25°C to 35°C; at 40°C, almost no kinase activity was detected. The activity of a holoprotein sample incubated at 40°C was nearly completely restored when the temperature returned to 25°C. UV/visible spectroscopy indicated that the protein was not denatured up to 45°C. At 50°C, however, Pfr denatured faster than the dark-adapted sample containing the Pr form of Agp1. The Pr visible spectrum was unaffected by temperatures of 20–45°C, whereas irradiated samples exhibited a clear temperature effect in the 30–40°C range in which prolonged irradiation resulted in the photoconversion of Pfr into a new spectral species termed Prx. Pfr to Prx photoconversion was dependent on the His-kinase module of Agp1; normal photoconversion occurred at 40°C in the mutant Agp1-M15, which lacks the C-terminal His-kinase module, and in a domain-swap mutant in which the His-kinase module of Agp1 is replaced by the His-kinase/response regulator module of the other *A. tumefaciens* phytochrome, Agp2. The temperature-dependent kinase activity and spectral properties in the physiological temperature range suggest that Agp1 serves as an integrated light and temperature sensor in *A. tumefaciens*.

## Introduction

In order to respond to environmental stimuli, bacteria utilize two-component regulatory systems that consist of His kinase sensors and cognate response regulators [1]. In *Agrobacterium tumefaciens*, the ability to infect plants by Ti DNA transformation is dependent on the *vir* genes, which are regulated by the VirA-VirG two-component regulatory system [2]. The expression of the *vir* genes is down-regulated at elevated temperature [3], an effect partly due to the autokinase activity of VirA, which decreases upon temperature increases from 28°C to over 32–37°C [3]. Here we describe a similar temperature effect for the His kinase of *A. tumefaciens* phytochrome Agp1.

Phytochromes are widely distributed photoreceptors found in plants, bacteria, and fungi that are most sensitive in the red and far-red regions of the visible spectrum [4]. Most phytochromes exhibit red/far-red photoreversibility; the chromophore adducts are converted by light between the red-absorbing form (Pr) and a far red-absorbing form (Pfr) with an absorption maximum that is increased by ∼50 nm [4]. Typically, phytochromes have an N-terminal chromophore module that contains PAS (“Per/Arnt/Sim”), GAF (“cGMP phosphodiesterase/adenylate cyclase/FhlA”), and PHY (“phytochrome”) core domains. The bilin chromophore of phytochromes becomes covalently linked to the protein during an autocatalytic lyase reaction. Typical bacterial and fungal phytochromes incorporate biliverdin as a natural chromophore with a vinyl ring A-side chain, while phycocyanobilin and phytochromobilin (ring A ethylidine side chain) are used by cyanobacteria and plants, respectively [5]. The biliverdin binding site is a conserved Cys at the N-terminus of the PAS domain, whereas phycocyanobilin- and phytochromobilin-binding phytochromes have a chromophore-binding Cys in the GAF domain at the center of the chromophore module [5]. The three-dimensional structures of the chromophore modules of bacterial phytochromes indicate that the GAF domain forms the tightest contact with the chromophore. The PAS and GAF domains are connected in a knotted structure, and the PHY domain forms a tongue-like structure that folds back on the chromophore [6]–[9].

In most bacterial and fungal phytochromes, including Agp1 from *A. tumefaciens*, the N-terminal chromophore module is linked to a C-terminal His kinase that is most likely connected to the PHY domain by a long helix; there seem to be no other contacts between the N- and C-terminal modules [7], [10], [11]. His kinase activity is most often modulated by light, although different Pr/Pfr patterns have been identified. Cph1, the first characterized prokaryotic phytochrome from the cyanobacterium *Synechocystis* PCC6803, exhibits strong autophosphorylation activity in the Pr form. This activity is down-regulated to ∼20% upon red irradiation, which triggers photoconversion into Pfr [12], [13]. The same principle holds for other cyanobacterial phytochromes [14] and for Agp1 [15]. In BphP of *Pseudomonas aeruginosa*, the His kinase activity did not differ significantly between the Pr and the Pfr forms [16], whereas for *P. syringae* phytochrome [17] and for *A. tumefaciens* phytochrome AtBphP2 (Agp2) [18], strong Pfr activity and weak Pr activity have been reported. This pattern also applies to FphA from the fungus *Aspergillus nidulans* [19]. Ppr, a chimeric photoreceptor from *Rhodospirillum centenum* in which an N-terminal photoactive yellow protein is linked with a phytochrome, displays His kinase activity in the dark that is up-regulated by light [20]. Strong Pr activity and weak Pfr activity dominate among the prototypical bacterial phytochromes, but phosphorylation activity cannot be switched off completely by light, and residual activity has been attributed to residual Pr under saturating red irradiation. Use of a locked 15*Ea* chromophore allows the generation of an Agp1 adduct that exists completely in the Pfr state and exhibits His kinase activity [15]. It thus appears that Agp1 has kinase activity in both the Pr and the Pfr states. Since light modulates kinase activity by a factor of 3–5 only, the impression arises that the kinase activity is not the only parameter which is important for signaling or that the activity might be modulated by other environmental stimuli in addition to light. Our present investigation of the effects of temperature on Agp1 kinase activity provide evidence in support of the latter possibility. We also found that the spectral properties of Agp1 are affected by temperature, and that these temperature effects are mediated through the His-kinase module.

## Results

**Figure 1:**
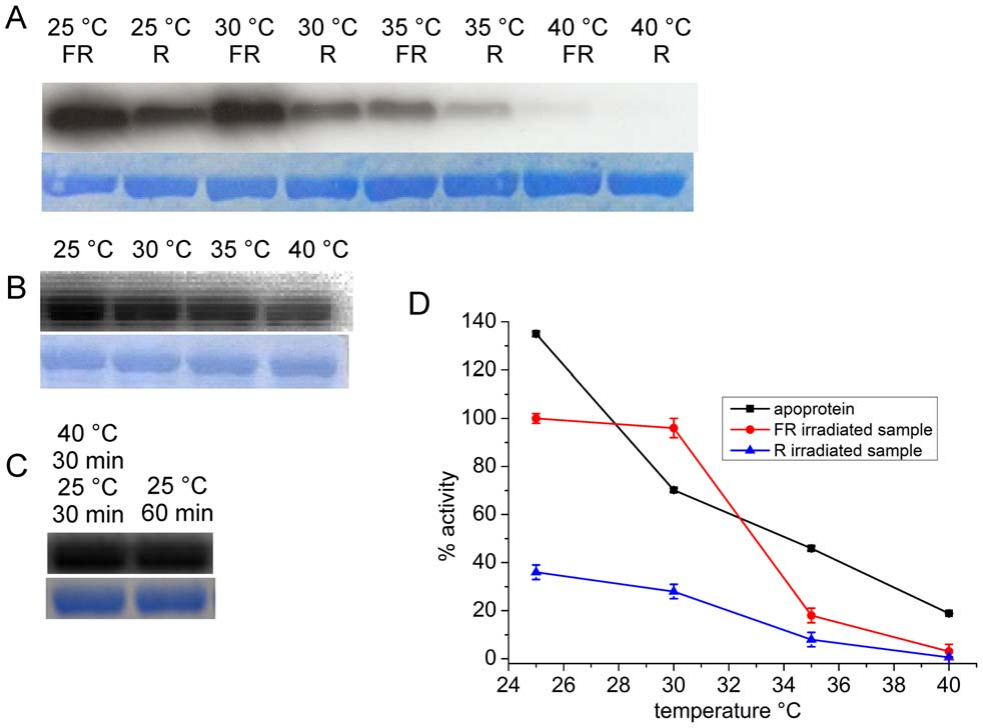
Autophosphorylation of Agp1 at various temperatures. (A) Autoradiogram (above) and Coomassie-stained blot (below) of Agp1 holoprotein. Samples were irradiated either with far-red (FR) or red (R) light and incubated with γ-^32^P ATP in darkness at the indicated temperatures. (B) Autoradiogram (above) and Coomassie-stained blot (below) of Agp1 apoprotein incubated with γ-^32^P ATP at the indicated temperatures. (C) Autoradiogram (above) and Coomassie-stained blot (below) of FR-treated holoprotein samples. Before incubation with γ-^32^P ATP in darkness at 25°C, one sample was kept at 25°C for 60 min (right lane); the other sample (left lane) was kept at 40°C for 30 min and then shifted to 25°C for 30 min. (D) Mean phosphorylation intensities of three experiments as shown in (A) and (B). Error bars denote standard error. The mean intensity of the FR-treated holoprotein at 25°C was set to 100%; at this temperature, the signal intensity of the apoprotein was 1.2-fold higher.

During a series of phosphorylation experiments with wild-type Agp1 and various Agp1 mutants, we observed that continuous irradiation with light-emitting diodes had a strong and unexpected effect on autophosphorylation (data not shown). Since the light-emitting diodes increased the sample temperature by several degrees, we tested for a possible temperature effect on Agp1 autophosphorylation. In subsequent experiments, Agp1 was irradiated before the incubation with labeled ATP at various temperatures. After far-red pre-irradiation, the major protein fraction is in the Pr form, whereas pre-irradiation with red light adjusts ∼90% Pfr [15]. The strongest autophosphorylation signal was obtained for the far red-treated sample at 25°C (Figure 1A). This signal was set to 100%; the signal from the red-irradiated sample was 36% (Figure 1D). The 3∶1 ratio of far-red/red-treated samples is qualitatively in accordance with earlier studies in which a 5∶1 ratio was observed under slightly different conditions [15], [21]. Similar results were obtained for far red- and red-pretreated samples at 30°C (Figure 1A). At 35°C, the autophosphorylation of the far red- and red-irradiated samples was very weak (18% and 8%, respectively) and at 40°C there was almost no autophosphorylation detected (Figure 1A).

We also tested the temperature effect on autophosphorylation activity of the apoprotein. At 25°C, apoprotein phosphorylation was 1.2±0.1-fold higher than that of Pr, again in accordance with earlier studies [15]. Autophosphorylation also decreased with increasing temperature (Figure 1B), but the pattern differed from that of the Pr or Pfr forms. From 25°C to 30°C the apoprotein activity decreased to a larger extent than the holoprotein, whereas the apoprotein displayed higher activity at 35–40°C than the corresponding Pr sample (Figure 1D).

For the Pr form, we also investigated whether the loss of activity upon temperature increase was regained following a subsequent temperature decrease. To this end, we incubated far red-treated Agp1 at 40°C for 30 min, shifted the sample to 25°C, and evaluated autophosphorylation versus a control sample maintained at 25°C (Figure 1C). The autophosphorylation activity of the temperature treated sample was 85±1% of the control, indicating that the temperature increase did not result in irreversible denaturation of the His kinase domain.

**Figure 2:**
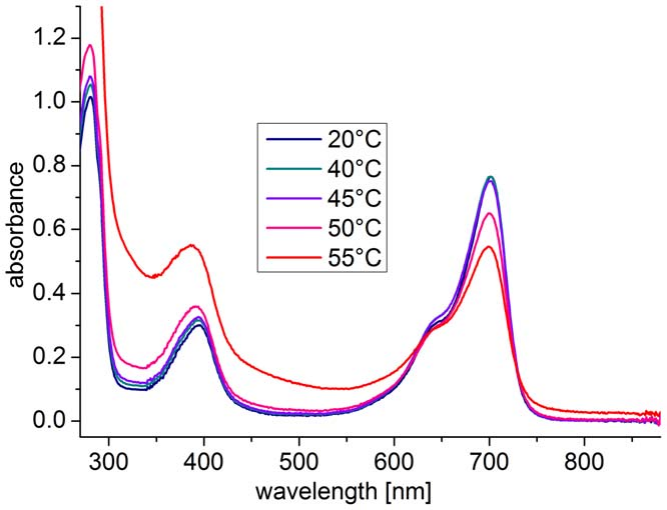
Absorption spectra of the Pr form of Agp1 at various temperatures.

Although the UV/visible spectral properties of Agp1 have been measured in various temperature ranges, such studies have not been performed above 30°C. We therefore investigated the spectral properties of Agp1 over a broad temperature range. We initially measured the spectra of dark-adapted Agp1, in which the holoprotein is in the Pr form, several times; the temperature between two subsequent scans was increased by 5°C to cover a range from 5°C to 60°C (data from 20–55°C appear in Figure 2). After each temperature increase, the sample in the cuvette was incubated for 30 min before the next measurement started. We detected no substantial impact of temperature on the spectra between 5°C and 40°C. At 45°C, a decrease in the absorbance in the 700 nm range was accompanied by a slight increase at lower wavelengths (Figure 2). This absorbance increase, which is characteristically more pronounced at shorter wavelengths, is due to scattering (indicative of protein denaturation), whereas the decrease in the 700-nm range indicates that the spectral properties of Pr are affected at this temperature. As expected, this effect increased with increasing temperature (Figure 2 and data not shown). Prolonged incubation at 60°C and above resulted in sedimentation of the protein in the cuvette. The scattering effect was not reversible when the temperature was decreased again.

**Figure 3:**
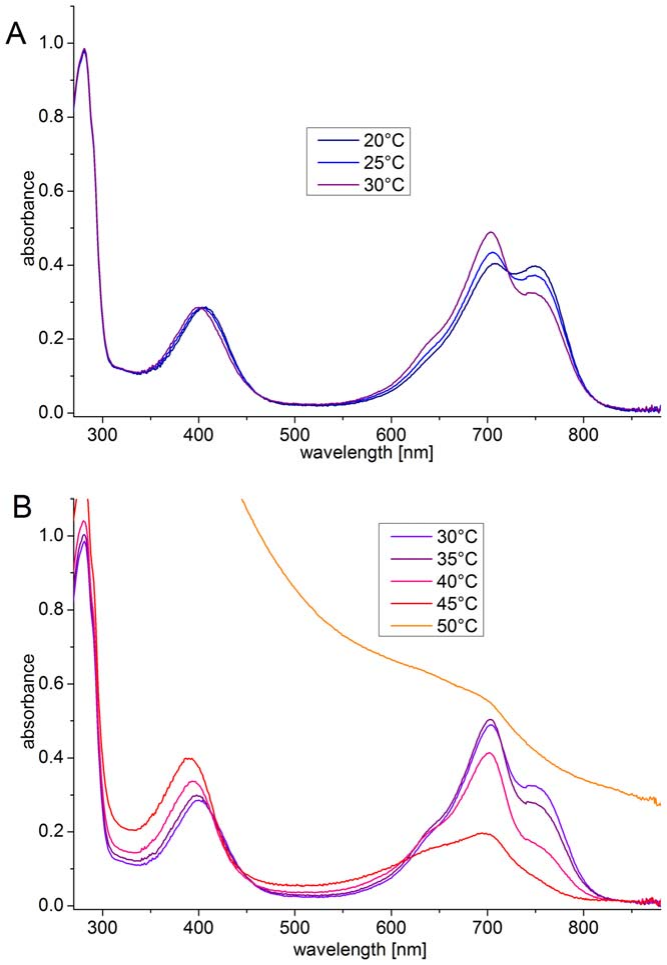
Absorption spectra of red-irradiated Agp1 at various temperatures. (A) Spectra in the 20°C to 30°C range. (B) Spectra in the 30°C to 50°C range. Before each scan, the sample was irradiated for 20 min with red light (655 nm; 20 µmol m^−2^ s^−1^).

**Figure 4:**
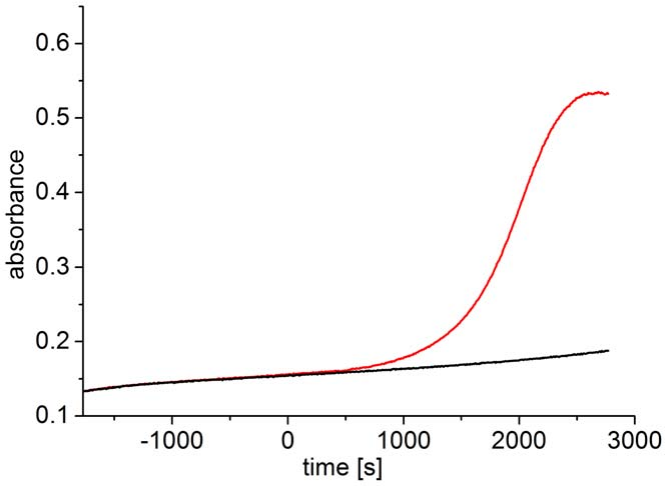
Scattering increase of dark-adapted and red-irradiated Agp1 at 50°C. Dark-adapted Agp1 was placed in a 50-°C cuvette and absorption was continuously monitored at 500 nm. One sample (red line) was irradiated with red light (655 nm, 20 µmol m^−2^ s^−1^) starting at t = 0. The other sample (black line) was kept in darkness.

In a similar set of experiments, red-irradiated Agp1 was scanned at various temperatures by raising the temperature in 5-°C steps and incubating for 30 min after the temperature adjustment. Before each scan the sample was irradiated for 20 min to approximate an equilibrium situation (see below). The scattering of red-irradiated Agp1 was not affected up to 40°C (Figure 3), but the 45°C sample showed slightly elevated scattering, and strong scattering occurred at 50°C, demonstrating that photoconverted Agp1 denatures at a lower temperature than the non-irradiated sample. To confirm this difference, we followed the scattering increases of two dark-adapted samples at 50°C; for one sample, red light was switched on during the measurement. After light onset, the subsequent scattering increase was substantially faster than that before the light treatment or that of the dark control (Figure 4). Thus, temperature-induced aggregation and denaturation are more pronounced in the photoconverted state than in the Pr ground state.

**Figure 5:**
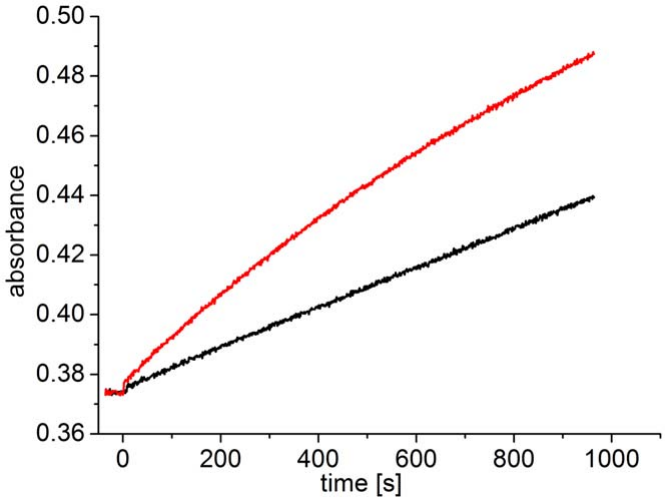
Dark reversion of Agp1 at 20°C (black) and 30°C (red) monitored at 700 nm. Each sample was irradiated with red light until no more absorption changes were evident. At t = 0, the red light was switched off.

In contrast to dark-adapted Agp1, the spectra of red-irradiated Agp1 are also affected by temperatures below 40°C. Two ranges, from 20°C to 30°C and from 30°C to 40°C, were distinguishable (Figures 3A and 3B, respectively). A temperature increase from 20°C to 30°C resulted in a decrease of absorption around 750 nm (the Pfr maximum) and an absorption increase around 700 nm (the Pr maximum). Thus, this temperature effect results simply from a change in the Pfr/Pr ratio. This ratio is determined by the two rates of photoconversion (Pr to Pfr and Pfr to Pr), by the rate of dark conversion, by the wavelength of actinic light and, if dark conversion plays a role in the process, by the intensity of actinic light. We measured the initial rates of photoconversion for both directions with 655 nm and 780 nm actinic light at 20°C and 30°C, and detected no substantial differences at either temperature (data not shown). However, we observed an enhanced rate of dark conversion at 30°C versus 20°C (Figure 5), leading us to conclude that the temperature effect on the Pr/Pfr ratio is due to a temperature effect on dark conversion.

**Figure 6:**
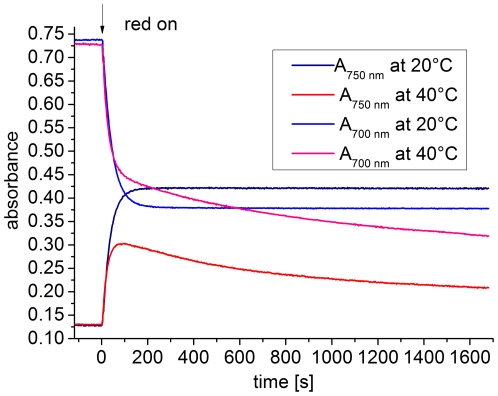
Photoconversion of Agp1 at 20°C and 40°C. Measurements were performed at 700 nm and 750 nm. At t = 0, red light (655 nm, 20 µmol m^−2^ s^−1^) was switched on.

Absorption at 750 nm decreased with increasing temperature in the 30–40°C range, but the absorption in the 700 nm range increased only slightly (30–35°C) or decreased (35–45°C; Figure 3B), suggesting that Pfr is spectrally affected or photoconverted into another, spectrally distinct form. We measured the course of absorption (700 nm and 750 nm, the absorption maxima of Pr and Pfr, respectively) of dark-adapted Agp1 during actinic red irradiation at 20°C and 40°C (Figure 6). The kinetic data measured at 20°C could be fitted to monoexponential functions (data not shown), indicative of a normal Pr to Pfr photoconversion [22]. Absorption changes at 40°C initially followed those seen at 20°C, again indicating normal Pr to Pfr photoconversion; however, after 100 s, these curves differed substantially from the 20°C curves (Figure 6). The absorbance values at 700 nm decreased continuously and approached lower final levels than the 20°C values, as implied by the above spectral measurements. Between 100 s and 700 s, the absorbance values at 700 nm of the 40°C sample were higher than those of the 20°C sample (Figure 6). This observation reflects the formation of the new spectral species by photoconversion from Pfr; the loss of absorbance at 700 nm due to Pr to Pfr conversion is partially compensated by the increase due to conversion from Pfr to the new form, which absorbs more strongly at 700 nm than does Pfr. The measurements at 750 nm are consistent with these interpretations, as the initial rise of absorption suggests normal Pr to Pfr photoconversion. The values maximized after ∼120 s and decreased thereafter (Figure 6). In this phase, the absorbances at 750 nm are determined by the photoconversion of Pfr to the new form, which we name Prx.

It is remarkable that at 40°C, absorption changes still continue after 30 min; photoequilibrium is not reached during that time (Figure 6), whereas spectral changes are nearly complete after 120 s and an apparent equilibrium is reached after ∼300 s at 20°C. We conclude that photoconversion from Pfr to Prx is inefficient, and that photoconversion of Prx to either Pfr or Pr is even less efficient (or zero), since otherwise the absorption losses by conversion into Prx would be offset by absorption increases during Pr formation (directly or via Pfr). Thus, the 40°C spectrum measured after continuous irradiation is largely determined by Prx. Although the pure spectrum of Prx cannot be precisely estimated with the available information, it is clear that it resembles the Pr spectrum but with a substantially smaller extinction coefficient. In this respect, Prx resembles the Pbl form of Avp1, a phytochrome from *A. vitis*, which is closely related to *A. tumefaciens* [23]. Avp1 has a normal Pr dark form, but two spectrally different species are formed upon photoconversion, Pfr and Pbl, which do not and do convert back to Pr in darkness, respectively.

**Figure 7:**
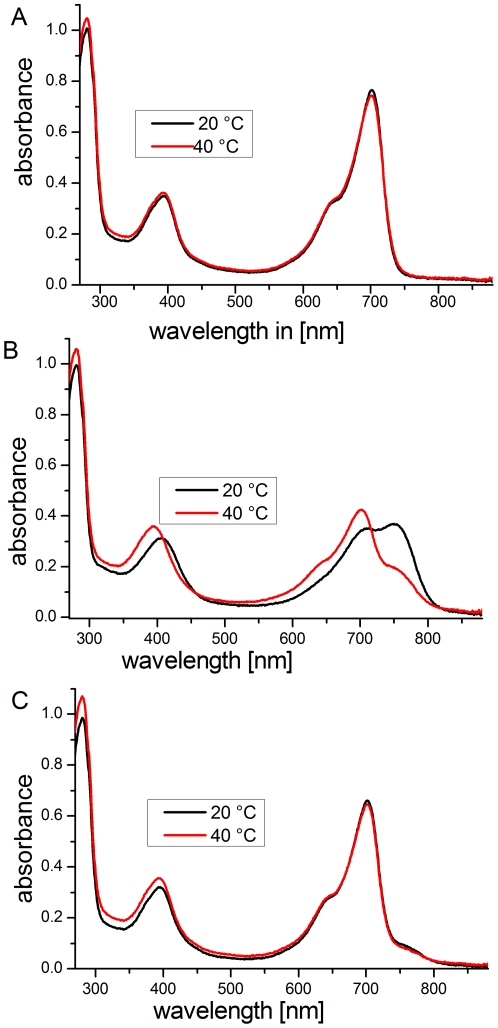
Absorption spectra of Agp1 during and after incubation at 20°C (black) or 40°C (red). (A) Non-irradiated Agp1 at 20°C and 40°C. (B) After red irradiation (20 min; 655 nm; 20 µmol m^−2^ s^−1^). (C) Both measurements are performed at 20°C after saturating far-red irradiation.

Next, we tested whether the temperature effect on spectral properties is reversible. Dark-adapted Agp1 (Figure 7A) was red-irradiated at 40°C (Figure 7B), brought to 20°C, and irradiated with far-red light to achieve high Pr levels (Figure 7C). The sample was compared with a control that was maintained at 20°C but otherwise treated identically. The final spectra of the two samples were nearly identical in the long wavelength range. In the shorter wavelength region, the transiently heated sample displayed slightly elevated scattering (Figure 7C), indicating that Prx formation is completely reversible and is not the result of irreversible denaturation. Prx reverted back to Pr in darkness (Figure 7 and data not shown).

**Figure 8:**
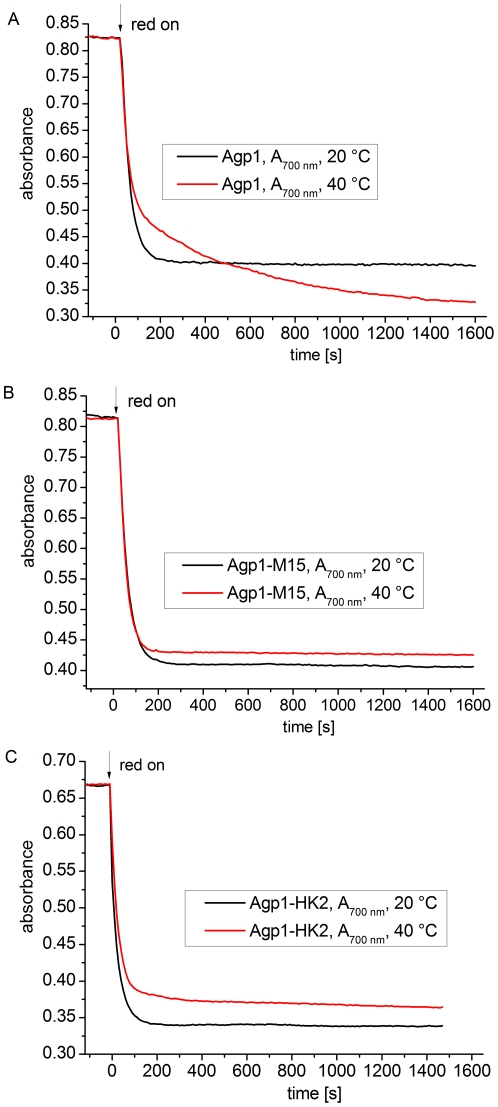
Photoconversion of Agp1, Agp1-M15, and Agp1-HK2 at 20°C and 40°C. Measurements were performed at 700 nm. At t = 0, red light (655 nm, 20 µmol m^−2^ s^−1^) was switched on.

We then investigated whether the formation of Prx is dependent on the presence of the His kinase. We compared time-drive measurements under continuous red irradiation of the full-length protein (Figure 8A) with a truncation mutant of Agp1 (Agp1-M15) that lacks the His kinase (Figure 8B). We also evaluated a new domain swap mutant, Agp1-HK2, in which the His-kinase module of Agp1 was replaced by the His-kinase/response regulator module of Agp2. In Agp1-M15, the absorption at 20°C and 40°C reached stable values after ∼300 s, and the curves were similar to each other (Figure 8B), indicating that no Prx is formed in this case. The slight difference in final absorption values between 20°C and 40°C was due to different dark conversion rates. Agp1-HK2 behaved similarly to Agp1-M15 (Figure 8C), although the absorption at 40°C decreased slightly in the later time range as well. We conclude that Prx formation at 40°C is dependent on the presence of the proper His kinase.

## Discussion

Nearly all chemical and biological processes are dependent on temperature, and thus it is not unexpected to discover that autophosphorylation of the *A. tumefaciens* phytochrome Agp1 is also modulated by temperature. However, typical enzyme activities increase with temperature and have a temperature optimum at physiologically high temperatures; the activity of the Agp1 His kinase decreases with increasing temperature within the physiological range (Figure 1). We investigated the kinase activity of Agp1 from 25–40°C and observed an optimum kinase-activity temperature at 25°C. The negative relationship between temperature and kinase activity points to a specific role of Agp1 as a temperature sensor.

VirA, another His kinase from *A. tumefaciens*, displays a similar temperature pattern. These VirA temperature effects are responsible for the temperature dependency of *vir* gene expression and bacterial virulence; infection rates at 35°C or 40°C are drastically lower than at 25°C. However, when the VirG response regulator of *A. tumefaciens* was replaced by a constitutively active VirG mutant expressing the *vir* genes at elevated temperatures, plant infection was still impaired [3]. Thus, additional temperature inhibition of virulence (in the bacterium or in the plant) must be considered. An active Agp1 His kinase may be required for infection, and loss of Agp1 activity may cause the loss of virulence. In accordance with this assumption, an *agp1^−^/agp2^−^* double mutant and an *agp1^−^* single mutant of *A. tumefaciens* exhibited impaired tumor induction in an *Arabidopsis thaliana* root infection assay ([24] and Rottwinkel, Sprissler, Lamparter, unpublished observation). The role of Agp1 in plant infection is presently under investigation.

Although the kinase activities of bacterial phytochromes have not been measured at different temperatures [12], [14]–[16] (but see [25] for the phytochrome-like protein CikA), links between phytochromes and temperature effects have been demonstrated. The *P. aeruginosa* phytochrome seems to be involved in stress response and quorum sensing, as heat tolerance was impaired in a phytochrome knockout mutant versus wild type [26]. In seed plants, phytochromes are important for temperature adaptations [27], with seed germination providing a classic example [28]–[30]. In *A. thaliana*, which has five phytochromes (*PhyA* to *PhyE*), the effect of temperature on germination has been investigated in wild-type plants and in double and triple *phyA*, *phyB*, and *phyE* mutants. The germination/temperature pattern differed from the wild-type pattern in most mutants [31]. The *HFR1* and *PIF4* transcription factors play a central role in temperature adaptation of *A. thaliana*, motivating a recent investigation of temperature effects on hypocotyl elongation in *hfr1*-, *pif4*-, and *phyB* mutants [32]. These mutants also displayed temperature responses that differed from the wild-type response, in line with a function of PhyB as temperature sensor. Plant phytochromes have a His kinase-like module in which His phosphorylation activity was lost during evolution [33], [34]; retention of this module may be explained by a temperature-sensing function.

In addition to VirA, the large group of His kinases also includes other proteins that serve as thermosensors [35]. For example, Hik33 (the *sll0698* gene product) from *Synechocystis* PCC6803, participates in the responses to cold stress and osmotic stress [36], and the plant pathogen *P. syringae* harbors the thermosensor CorS [37]. DesK is a thermo-sensitive His kinase from *Bacillus subtilis* with high autophosphorylation activity at 25°C and low activity at 37°C, a behavior comparable to the behaviors of VirA [38] and Agp1.

Several phytochrome-like proteins are known to exhibit temperature-dependent spectral properties in the physiological range. The cyanobacteriochrome Tlr0924 from *Thermosynechococcus elongatus* [39] is a blue-green photoreversible biliprotein with the GAF domain common to phytochromes; the spectral properties and dark conversion of this biliprotein are clearly temperature-dependent. SyB-Cph1, a phytochrome-like protein from the thermophilic cyanobacterium *Synechococcus* OS-B, displays faster photoconversion and faster dark conversion at higher temperatures [40]. The bacteriophytochrome BphP3 from *Bradyrhizobium* ORS278 utilizes phycocyanobilin as a chromophore, most likely as a result of horizontal gene transfer. This unusual phytochrome adopts a P-orange ground state with an absorption maximum of ∼600 nm, photoconverting into a Pr form with an absorption maximum of ∼670 nm. In darkness, equilibrium between P-orange and Pr is established with the fraction of each form being strongly temperature-dependent, and the absorption maximum of P-orange shifts to the blue range upon temperature increase.

To date, however, our observation of the temperature-dependent photoconversion of Pfr into Prx has not been reported, although photoproducts that are formed from Pr and are spectrally similar to Prx have been described. Bacteriophytochrome Avp1 from *A. vitis* with a Pr ground state photoconverts into two spectral forms, Pfr and Pbl [23], the latter of which resembles Prx. The chimeric PYP/phytochrome protein of *R. centenum* has a Pr ground state and photoconverts completely into a bleached form [20]. Bleached photoproducts have also been described for the D197A and H250A mutants of Agp1 [41], which are arrested in their photocycle due to impaired chromophore protonation. Prx could contain a deprotonated chromophore, as indicated for the Pbl form of Avp1 [23].

We assume that Prx formation at elevated temperatures reflects subtle protein conformational changes in the core of the chromophore module. These conformational changes are not the result of a direct temperature effect on protein conformation, but are mediated by the C-terminal His kinase. Thus, there are two directions of information flow in Agp1: light is sensed by the N-terminal chromophore module and transmitted to the His kinase, whereas temperature is sensed by the His kinase and communicated to the chromophore module. In plant phytochromes, a signal transduction pathway is initiated by a well-defined region in the chromophore module around the “knot” [42]. We suggest that Agp1 delivers information to other proteins through its N-terminal chromophore module. The temperature-induced protein conformational changes could then induce positive signals at elevated temperatures under conditions in which the His kinase is switched off.

## Materials and Methods

### Protein expression, purification, and assembly

All proteins used for these studies were expressed in *Escherichia coli* XL1-Blue cells containing the pAG1 [15], pAG1-M15 [43], or pAG1-HK2 expression vectors that encode full-length Agp1 protein, the chromophore module of Agp1, and the Agp1 domain-swap mutant Agp1-HK2, respectively. To construct pAG1-HK2, the pAG1 expression vector was modified as follows. The sequence encoding the C-terminal moiety of Agp2 containing the His kinase and the response regulator was amplified by PCR using Phusion polymerase (New England Biolabs). The expression vector pSA2 [44], modified as described in [45], was used as a template with the phosphorylated primers GAGCTTAATCACCGCGTCAAG and AATCCCAGCCATCAGCGCATC. Another PCR with non-phosphorylated primers (GCTTAATTAGCTGAGCTTGGACT and CTTATTGGTGCGCTGCAACTCC) amplified the entire pAG1 plasmid DNA except the sequence encoding the C-terminal His kinase. PCR products were purified by DNA electrophoresis and a DNA purification kit (Peqlab) as described by the manufacturer. The purified blunt-end PCR products were ligated with T4 ligase (New England Biolabs) overnight at 16°C [46] and transformed into the *E. coli* strain XL1-Blue. The DNA sequence of the new expression vector was checked by sequencing (GATC, Constance, Germany) and deposited in GenBank (JN003831).

All proteins used in this study contained a C-terminal His-tag for affinity purification. For recombinant expression, bacteria with the desired expression vector were grown in 1 L Luria-Bertani medium with ampicillin and tetracycline at 37°C until the cell density reached an OD_600_ of 0.6. Specific protein expression was induced with 50 µM isopropyl-1-thiol-ß-D-galactopyranoside. After ∼40 h of incubation at 18°C, the cell density reached an OD_600_ of ∼2.3. The cells were centrifuged at 5000×*g* for 10 min at 4°C, and the pellet was suspended in 20 mL extraction buffer (300 mM NaCl, 50 mM Tris/HCl, 5 mM EDTA, 10 mM DTT, pH 7.8). Cells were lysed using a French Pressure Cell Press (Aminco) with two passages at 20000 psi. The lysed cells were centrifuged at 20000×*g* for 30 min at 4°C. Proteins in the supernatant were precipitated with 50% ammonium sulfate, and the pellet was dissolved in wash buffer (300 mM NaCl, 50 mM Tris/HCl, 10 mM imidazole, pH 7.8), centrifuged again, and subjected to Ni^2+^-affinity chromatography (Qiagen, Hilden, Germany). The column was washed with 20 volumes of wash buffer, and apoprotein was eluted with concentrated imidazole buffer (300 mM NaCl, 50 mM Tris/HCl, 250 mM imidazole, pH 7.8). Protein was collected by 50% ammonium sulfate precipitation, resuspended in basic buffer (300 mM NaCl, 50 mM Tris/HCl, 5 mM EDTA, pH 7.8), and centrifuged. The apoprotein concentration was estimated from the absorption at 280 nm [15]. For apoprotein autophosphorylation assays, the protein was used at this stage of purification.

To obtain holoprotein, 50 µM DTT (final concentration) was added to the protein solution, and biliverdin (Frontier Scientific, Carnforth, UK) was added at 3-fold molar excess. Assembly at 20°C was monitored by UV/visible spectroscopy [15]. Excess free biliverdin was separated from the holoprotein using NAP-10 desalting columns (GE Healthcare) according to the manufacturer's instructions. Finally, the protein was dissolved and diluted in basic buffer. Details of protein expression and purification of Agp1 are also given in earlier publications [15], [47].

### Absorption spectra, rate of photoconversion, and dark reversion at various temperatures

Spectra were recorded with a Jasco V-550 photometer equipped with a temperature-controlled cuvette holder. For measurements at various temperatures, a delay time of 30 min was maintained before spectra measurement. During this delay, the temperature inside the cuvette reached the desired value, as monitored by a thermometer. For photoconversion measurements, the sample was irradiated directly in the photometer with a light-emitting diode of λ_max_ = 655 nm and an intensity of 20 µmol m^−2^ s^−1^. Spectra were recorded after 20 min of irradiation; for most conditions, photoequilibrium was reached after this time. For the rates of photoconversion, the absorptions at 700 nm and 750 nm were measured continuously during red irradiation. The onset of light was set as t = 0. For the rates of dark reversion, absorption values at 700 nm were measured continuously; samples were irradiated with red light until a photoequilibrium was apparently achieved, and then the light was switched off (t = 0). To follow protein denaturation over time, the cuvette was placed into the cuvette holder, the temperature inside the cuvette was adjusted to 50°C, and the absorption at 500 nm was continuously recorded. Phytochrome absorbs only weakly at this wavelength, and thus an absorbance increase is indicative of increased scattering. After a given time the sample was either irradiated with red light to induce photoconversion or left in the dark. To test the stability of Agp1 at 40°C, a sample of Agp1 in a cuvette was incubated inside the photometer at 40°C for 30 min, red-irradiated for 20 min, and brought to 20°C. After an adaptation time of 30 min, the sample was irradiated with far-red light for 20 min. Spectra were recorded at each point of the treatment. A control sample was treated in the same manner except for a constant temperature of 20°C.

### Phosphorylation

An earlier protocol [48] was adapted for evaluation of autophosphorylation. Holoprotein phosphorylation experiments were performed under a blue-green safelight (light-emitting diode of λ_max_ = 505 nm) or in darkness. The concentration of Agp1 was 1.2 mg/mL, an estimate based on the absorbance at 280 nm [15]. Each sample contained 5 µL Agp1 and was irradiated with either 655 nm red light from a light-emitting diode (20 µmol m^−2^ s^−1^) or with 780 nm far-red light from a light-emitting diode (80 µmol m^−2^ s^−1^). The irradiation time was always 2 min, and the temperature during this irradiation was 25°C. Directly after irradiation, 15 µL phosphorylation buffer (25 mM Tris/HCl, 5 mM MgCl_2_, 4 mM β-mercaptoethanol, 0.2 mM EDTA pH 7.8, 50 mM KCl, 5% ethylene glycol, 0.45 µM (50 MBq/ml) γ-^32^P ATP, pH 7.8) were added to each sample. After mixing, the samples were immediately transferred to a thermal block set to the given temperature for 30 min. The phosphorylation reaction was stopped by adding 10 µL loading buffer (30% glycerol, 6% SDS, 300 mM DTT, 0.01% bromphenol blue, 240 mM Tris/HCl, pH 6.7).

Ten microliters of each sample were loaded onto 10% SDS-PAGE gels. Following electrophoresis, the protein was transferred to a polyvinylidene fluoride membrane (Millipore) with a Trans-Blot Semi-Dry blot apparatus (Biorad). The dried membrane was exposed to a phosphoimager plate (Fuji) for ∼5 min, and quantification was performed with the fluorescent image analyzer FLA 2000 (Fuji) and integrated analysis software. In some cases, the polyvinylidene fluoride membrane was also exposed on X-ray film for 120 min. For Coomassie staining of the protein bands, the membrane was incubated with SimplyBlue SafeStain (Invitrogen) following the manufacturer's instructions, except that the membrane was briefly shaken in 50% methanol before staining. Apoprotein phosphorylation experiments were performed in the same manner except that the protein solution was not irradiated and all incubation steps occurred in the light. Direct comparisons of apoprotein and holoprotein at 25°C revealed a 1.2±0.1 apoprotein/holoprotein (far red-irradiated) ratio. This ratio was used to normalize the temperature-dependent activities of holoprotein and apoprotein, which were probed in different sets of experiments.

Phosphorylation was also tested after a transient temperature increase. To this end, far red-irradiated Agp1 was incubated at 40°C for 30 min, then returned to 25°C for another 30 min. After 60 min incubation, the phosphorylation assay was performed as described. A control sample was continuously kept at 25°C for 60 min.

## References

[1] Gao R, Stock AM (2009). Biological Insights from Structures of Two-Component Proteins.. Annual Review of Microbiology.

[2] Heath JD, Charles TC, Nester EW, Hoch J, Silhavy TJ (1995). Ti plasmid and chromosomally encoded two-component systems important in plant cell transformation by *Agrobacterium* species.. Two-component signal transduction.

[3] Jin S, Song YN, Deng WY, Gordon MP, Nester EW (1993). The regulatory VirA protein of *Agrobacterium tumefaciens* does not function at elevated temperatures.. J Bacteriol.

[4] Rockwell NC, Su YS, Lagarias JC (2006). Phytochrome structure and signaling mechanisms.. Annu Rev Plant Biol.

[5] Lamparter T (2004). Evolution of cyanobacterial and plant phytochromes.. FEBS Lett.

[6] Wagner JR, Brunzelle JS, Forest KT, Vierstra RD (2005). A light-sensing knot revealed by the structure of the chromophore-binding domain of phytochrome.. Nature.

[7] Yang X, Kuk J, Moffat K (2008). Crystal structure of *Pseudomonas aeruginosa* bacteriophytochrome: photoconversion and signal transduction.. Proc Natl Acad Sci U S A.

[8] Essen LO, Mailliet J, Hughes J (2008). The structure of a complete phytochrome sensory module in the Pr ground state.. Proc Natl Acad Sci U S A.

[9] Scheerer P, Michael N, Park JH, Nagano S, Choe HW (2010). Light-induced conformational changes of the chromophore and the protein in phytochromes: bacterial phytochromes as model systems.. ChemPhysChem.

[10] Li H, Zhang JR, Vierstra RD, Li HL (2010). Quaternary organization of a phytochrome dimer as revealed by cryoelectron microscopy.. Proc Natl Acad Sci U S A.

[11] Evans K, Grossmann JG, Fordham-Skelton AP, Papiz MZ (2006). Small-angle X-ray scattering reveals the solution structure of a bacteriophytochrome in the catalytically active Pr state.. J Mol Biol.

[12] Yeh KC, Wu SH, Murphy JT, Lagarias JC (1997). A cyanobacterial phytochrome two-component light sensory system.. Science.

[13] Esteban B, Carrascal M, Abian J, Lamparter T (2005). Light-induced conformational changes of cyanobacterial phytochrome Cph1 probed by limited proteolysis and autophosphorylation.. Biochemistry.

[14] Hübschmann T, Jorissen HJ, Börner T, Gärtner W, Tandeau de Marsac N (2001). Phosphorylation of proteins in the light-dependent signalling pathway of a filamentous cyanobacterium.. Eur J Biochem.

[15] Lamparter T, Michael N, Mittmann F, Esteban B (2002). Phytochrome from *Agrobacterium tumefaciens* has unusual spectral properties and reveals an N-terminal chromophore attachment site.. Proc Natl Acad Sci U S A.

[16] Tasler R, Moises T, Frankenberg-Dinkel N (2005). Biochemical and spectroscopic characterization of the bacterial phytochrome of *Pseudomonas aeruginosa*.. FEBS J.

[17] Bhoo SH, Davis SJ, Walker J, Karniol B, Vierstra RD (2001). Bacteriophytochromes are photochromic histidine kinases using a biliverdin chromophore.. Nature.

[18] Karniol B, Vierstra RD (2003). The pair of bacteriophytochromes from *Agrobacterium tumefaciens* are histidine kinases with opposing photobiological properties.. Proc Natl Acad Sci U S A.

[19] Brandt S, von Stetten D, Günther M, Hildebrandt P, Frankenberg-Dinkel N (2008). The Fungal Phytochrome FphA from *Aspergillus nidulans*.. J Biol Chem.

[20] Kyndt JA, Fitch JC, Seibeck S, Borucki B, Heyn MP (2010). Regulation of the Ppr histidine kinase by light-induced interactions between its photoactive yellow protein and bacteriophytochrome domains.. Biochemistry.

[21] Inomata K, Hammam MAS, Kinoshita H, Murata Y, Khawn H (2005). Sterically locked synthetic bilin derivatives and phytochrome Agp1 from *Agrobacterium tumefaciens* form photoinsensitive Pr- and Pfr-like adducts.. J Biol Chem.

[22] Mancinelli A, Kendrick RE, Kronenberg GHM (1994). The physiology of phytochrome action.. Photomorphogenesis in Plants, 2nd edition.

[23] Rottwinkel G, Oberpichler I, Lamparter T (2010). Bathy phytochromes in rhizobial soil bacteria.. J Bacteriol.

[24] Rottwinkel G (2011).

[25] Mutsuda M, Michel KP, Zhang X, Montgomery BL, Golden SS (2003). Biochemical properties of CikA, an unusual phytochrome-like histidine protein kinase that resets the circadian clock in Synechococcus elongatus PCC 7942.. J Biol Chem.

[26] Barkovits K, Harms A, Benkartek C, Smart JL, Frankenberg-Dinkel N (2008). Expression of the phytochrome operon in *Pseudomonas aeruginosa* is dependent on the alternative sigma factor RpoS.. FEMS Microbiol Lett.

[27] Franklin KA (2009). Light and temperature signal crosstalk in plant development.. Curr Opin Plant Biol.

[28] Scheibe J, Lang A (1969). Lettuce seed germination: effects of high temperature and of repeated far-red treatment in relation to phytochrome.. Photochem Photobiol.

[29] Payne PI, Dyer TA (1972). Phytochrome and temperature relations in *Lactuca sativa* L. Grand Rapids seed germination after thermo-dormancy.. Nat New Biol.

[30] Fielding A, Kristie DN, Dearman P (1992). The temperature dependence of Pfr action governs the upper temperature.. Photochem Photobiol.

[31] Heschel MS, Selby J, Butler C, Whitelam GC, Sharrock RA (2007). A new role for phytochromes in temperature-dependent germination.. New Phytol.

[32] Foreman J, Johansson H, Hornitschek P, Josse EM, Fankhauser C (2011). Light receptor action is critical for maintaining plant biomass at warm ambient temperatures.. Plant Journal.

[33] Schneider-Poetsch HA (1992). Signal transduction by phytochrome: phytochromes have a module related to the transmitter modules of bacterial sensor proteins.. Photochem Photobiol.

[34] Elich TD, Chory J (1997). Phytochrome: if it looks and smells like a histidine kinase, is it a histidine kinase?. Cell.

[35] Klinkert B, Narberhaus F (2009). Microbial thermosensors.. Cell Mol Life Sci.

[36] Mikami K, Kanesaki Y, Suzuki I, Murata N (2002). The histidine kinase Hik33 perceives osmotic stress and cold stress in Synechocystis sp PCC 6803.. Molecular Microbiology.

[37] Braun Y, Smirnova AV, Schenk A, Weingart H, Burau C (2008). Component and protein domain exchange analysis of a thermoresponsive, two-component regulatory system of *Pseudomonas* syringae.. Microbiology-Sgm.

[38] Albanesi D, Martin M, Trajtenberg F, Mansilla MC, Haouz A (2009). Structural plasticity and catalysis regulation of a thermosensor histidine kinase.. Proc Natl Acad Sci U S A.

[39] Rockwell NC, Njuguna SL, Roberts L, Castillo E, Parson VL (2008). A second conserved GAF domain cysteine is required for the blue/green photoreversibility of cyanobacteriochrome Tlr0924 from *Thermosynechococcus elongatus*.. Biochemistry.

[40] Ulijasz AT, Cornilescu G, von Stetten D, Kaminski S, Mroginski MA (2008). Characterization of two thermostable cyanobacterial phytochromes reveals global movements in the chromophore-binding domain during photoconversion.. J Biol Chem.

[41] von Stetten D, Seibeck S, Michael N, Scheerer P, Mroginski MA (2007). Highly conserved residues D197 and H250 in Agp1 phytochrome control the proton affinity of the chromophore and Pfr formation.. J Biol Chem.

[42] Nagatani A (2010). Phytochrome: structural basis for its functions.. Curr Opin Plant Biol.

[43] Scheerer P, Michael N, Park JH, Noack S, Förster C (2006). Crystallization and preliminary X-ray crystallographic analysis of the N-terminal photosensory module of phytochrome Agp1, a biliverdin-binding photoreceptor from *Agrobacterium tumefaciens*.. J Struct Biol.

[44] Lamparter T, Michael N (2005). *Agrobacterium* phytochrome as an enzyme for the production of ZZE bilins.. Biochemistry.

[45] Inomata K, Noack S, Hammam MAS, Khawn H, Kinoshita H (2006). Assembly of synthetic locked chromophores with *Agrobacterium* phytochromes Agp1 and Agp2.. J Biol Chem.

[46] Sambrook J, Russell WD (2001). Molecular Cloning..

[47] Lamparter T, Carrascal M, Michael N, Martinez E, Rottwinkel G (2004). The biliverdin chromophore binds covalently to a conserved cysteine residue in the N-terminus of *Agrobacterium phytochrome* Agp1.. Biochemistry.

[48] Noack S, Lamparter T, Melvin IS (2007). Light modulation of histidine-kinase activity in bacterial phytochromes monitored by size exclusion chromatography, crosslinking, and limited proteolysis.. Methods in Enzymology, Two-Component Signaling Systems, Part B.

